# Prediction of postoperative stroke in patients experienced coronary artery bypass grafting surgery: a machine learning approach

**DOI:** 10.3389/fcvm.2024.1448740

**Published:** 2024-12-13

**Authors:** Shiqi Chen, Kan Wang, Chen Wang, Zhengfeng Fan, Lizhao Yan, Yixuan Wang, Fayuan Liu, JiaWei Shi, QianNan Guo, NianGuo Dong

**Affiliations:** ^1^ Department of Cardiovascular Surgery, Union Hospital, Tongji Medical College, Huazhong University of Science and Technology, Wuhan, Hubei, China; ^2^ Department of Hand Surgery, Union Hospital, Tongji Medical College, Huazhong University of Science and Technology, Wuhan, Hubei, China

**Keywords:** coronary artery bypass grafting (CABG), machine learning (ML), preoperative clinical features, postoperative complications, stroke, random forest

## Abstract

**Background:**

Coronary artery bypass grafting (CABG) surgery has been a widely accepted method for treating coronary artery disease. However, its postoperative complications can have a significant effect on long-term patient outcomes. A retrospective study was conducted to identify before and after surgery that contribute to postoperative stroke in patients undergoing CABG, and to develop predictive models and recommendations for single-factor thresholds.

**Materials and methods:**

We utilized data from 1,200 patients who undergone CABG surgery at the Wuhan Union Hospital from 2016 to 2022, which was divided into a training group (*n* = 841) and a test group (*n* = 359). 33 preoperative clinical features and 4 postoperative complications were collected in each group. LASSO is a regression analysis method that performs both variable selection and regularization to enhance model prediction accuracy and interpretability. The LASSO method was used to verify the collected features, and the SHAP value was used to explain the machine model prediction. Six machine learning models were employed, and the performance of the models was evaluated by area under the curve (AUC) and decision curve analysis (DCA). AUC, or area under the receiver operating characteristic curve, quantifies the ability of a model to distinguish between positive and negative outcomes. Finally, this study provided a convenient online tool for predicting CABG patient post-operative stroke.

**Results:**

The study included a combined total of 1,200 patients in both the development and validation cohorts. The average age of the participants in the study was 60.26 years. 910 (75.8%) of the patients were men, and 153 (12.8%) patients were in NYHA class III and IV. Subsequently, LASSO model was used to identify 11 important features, which were mechanical ventilation time, preoperative creatinine value, preoperative renal insufficiency, diabetes, the use of an intra-aortic balloon pump (IABP), age, Cardiopulmonary bypass time, Aortic cross-clamp time, Chronic Obstructive Pulmonary Disease (COPD) history, preoperative arrhythmia and Renal artery stenosis in descending order of importance according to the SHAP value. According to the analysis of receiver operating characteristic (ROC) curve, AUC, DCA and sensitivity, all seven machine learning models perform well and random forest (RF) machine model was found to perform best (AUC-ROC = 0.9008, Accuracy: 0.9008, Precision: 0.6905; Recall: 0.7532, F1: 0.7205). Finally, an online tool was established to predict the occurrence of stroke after CABG based on the 11 selected features.

**Conclusion:**

Mechanical ventilation time, preoperative creatinine value, preoperative renal insufficiency, diabetes, the use of an intra-aortic balloon pump (IABP), age, Cardiopulmonary bypass time, Aortic cross-clamp time, Chronic Obstructive Pulmonary Disease (COPD) history, preoperative arrhythmia and Renal artery stenosis in the preoperative and intraoperative period was associated with significant postoperative stroke risk, and these factors can be identified and modeled to assist in implementing proactive measures to protect the brain in high-risk patients after surgery.

## Introduction

Coronary heart disease (CHD) is a major contributor to the global burden of cardiovascular diseases, with an estimated 125 million sufferers worldwide (1). Coronary artery disease (CAD) may result in a range of cardiovascular complications, including myocardial infarction ischemic cardiomyopathy, and culminating in heart failure (HF) (2). In the United States alone, costs associated with heart failure total more than $40 billion per year, posing an ever-increasing financial strain on individuals, families and the healthcare system (3).

Over recent years, coronary artery bypass grafting (CABG) surgery has become increasingly widespread, with more than 350,000 operations conducted in the United States (4). Advances in technology have enabled CABG to be used for an even wider range of patients than prior to its increased availability (5). Despite these improvements, there is still the inherent risks of postoperative complications. Al-Ruzzeh S and his colleagues identified a range of preoperative risk factors such as cerebrovascular accident, chronic obstructive pulmonary disease, diabetes, smoking history, hypertension and peripheral vascular disease that can raise the likelihood of postoperative complications (6). Eagle and colleagues highlighted that postoperative complications account for 10% of surgical expenses (7). It is vitally important therefore to predict the risk of postoperative complications using preoperative risk factors in order to facilitate earlier intervention. To this end, it is suggested that preoperative risk factors can be used to anticipate and intervene against postoperative complications of CABG.

Recently, Artificial intelligence (AI) is a hot topic in the medical field. Powerful AI technology can unlock important information hidden in massive clinical data and make contributions to clinical decision-making (8). In this study, six machine learning models and Logistic Regression model were applied to analyze the correlation between preoperative risk factors and postoperative stroke in 1,200 patients who underwent CABG surgery. Results suggest that AI could be a useful tool to uncover hidden information in clinical data and optimize the prognosis and treatment of CABG patients. This can assist us in making more active interventions for patients at risk of stroke following surgery.

## Materials and methods

### Study population and data source

A total of 1,200 patients who underwent CABG surgery in the Department of Cardiology Surgery of the Wuhan Union hospital between January 2016 and June 2020 were randomly divided into two groups: one training group included 7/10 of the total sample, while the other testing group included 3/10 of the sample. Complications that occurred within a 30-day period following the surgical procedure were considered as postoperative complications. Postoperative stroke refers to a sudden loss of local or total cerebral nerve function, either ischemic or hemorrhagic, that occurs during or after surgery and is characterized by vasogenic effects.

Clinical information from these two groups was recorded and is presented in Table 1. All surgeries for CABG were performed by cardiac surgery specialist to ensure quality in our hospital, like Deputy Chief Physician or above. This study belongs to single centered retrospective research. This study is reported in line with the STROCSS guidelines (9, 10).

**Table 1:** Datasets were divided into training and testing sets (7:3).

	Level	Overall	Train	Test	*P*-value
Numbers (*n*)		1,200	841	359	
Gender (*n*%)	Female	290 (24.2)	201 (23.9)	89 (24.8)	0.798
	Male	910 (75.8)	640 (76.1)	270 (75.2)	
Age (years) [mean (SD)]		60.26 (8.95)	60.17 (8.99)	60.49 (8.87)	0.563
Percutaneous coronary intervention (PCI) (*n*%)	No	1,046 (87.2)	727 (86.4)	319 (88.9)	0.294
	Yes	154 (12.8)	114 (13.6)	40 (11.1)	
Smoking (*n*%)	No	601 (50.1)	425 (50.5)	176 (49.0)	0.677
	Yes	599 (49.9)	416 (49.5)	183 (51.0)	
Numbers of stenosed coronary vessel (*n*%)	1	81 (6.8)	57 (6.8)	24 (6.7)	0.626
	2	237 (19.8)	160 (19.0)	77 (21.4)	
	3	882 (73.5)	624 (74.2)	258 (71.9)	
NYHA (*n*%)	0	1,047 (87.2)	720 (85.6)	327 (91.1)	0.063
	1	153 (12.8)	121 (14.4)	32 (8.9)	
Emergency (*n*%)	No	1,184 (98.7)	828 (98.5)	356 (99.2)	0.479
	Yes	16 (1.3)	13 (1.5)	3 (0.8)	
Reoperation (*n*%)	No	1,190 (99.2)	832 (98.9)	358 (99.7)	0.301
	Yes	10 (0.8)	9 (1.1)	1 (0.3)	
Cardiac arrest (*n*%)	No	771 (64.2)	539 (64.1)	232 (64.6)	0.912
	Yes	429 (35.8)	302 (35.9)	127 (35.4)	
Bridge number (*n*%)	1	54 (4.5)	40 (4.8)	14 (3.9)	0.896
	2	155 (12.9)	109 (13.0)	46 (12.8)	
	3	399 (33.2)	271 (32.2)	128 (35.7)	
	4	452 (37.7)	320 (38.0)	132 (36.8)	
	5	126 (10.5)	90 (10.7)	36 (10.0)	
	6	13 (1.1)	10 (1.2)	3 (0.8)	
	7	1 (0.1)	1 (0.1)	0 (0.0)	
Numbers of arterial anastomosis (*n*%)	0	205 (17.1)	139 (16.5)	66 (18.4)	0.885
	1	981 (81.8)	692 (82.3)	289 (80.5)	
	2	10 (0.8)	7 (0.8)	3 (0.8)	
	3	4 (0.3)	3 (0.4)	1 (0.3)	
Numbers of venous anastomosis (*n*%)	0	50 (4.2)	36 (4.3)	14 (3.9)	0.946
	1	115 (9.6)	84 (10.0)	31 (8.6)	
	2	365 (30.4)	251 (29.8)	114 (31.8)	
	3	471 (39.2)	327 (38.9)	144 (40.1)	
	4	179 (14.9)	128 (15.2)	51 (14.2)	
	5	19 (1.6)	14 (1.7)	5 (1.4)	
	6	1 (0.1)	1 (0.1)	0 (0.0)	
Cardiopulmonary bypass (CPB) time [mean (SD)]		40.38 (59.49)	40.49 (59.39)	40.11 (59.81)	0.919
Aortic cross-clamp time [mean (SD)]		23.50 (35.87)	23.44 (35.68)	23.65 (36.37)	0.926
Cardiotonic drugs (*n*%)	No	1,187 (98.9)	830 (98.7)	357 (99.4)	0.398
	Yes	13 (1.1)	11 (1.3)	2 (0.6)	
Hypertension (*n*%)	No	191 (15.9)	136 (16.2)	55 (15.3)	0.777
	Yes	1,009 (84.1)	705 (83.8)	304 (84.7)	
Diabetes mellitus (DM) (*n*%)	No	652 (54.3)	457 (54.3)	195 (54.3)	1.000
	Yes	548 (45.7)	384 (45.7)	164 (45.7)	
Hyperlipidemia (*n*%)	No	393 (32.8)	275 (32.7)	118 (32.9)	1.000
	Yes	807 (67.2)	566 (67.3)	241 (67.1)	
Preoperative creatinine value [mean (SD)]		85.11 (59.85)	83.88 (51.73)	88.00 (75.54)	0.275
Preoperative hemodialysis (*n*%)	No	1,193 (99.4)	837 (99.5)	356 (99.2)	0.737
	Yes	7 (0.6)	4 (0.5)	3 (0.8)	
Preoperative renal insufficiency (*n*%)	No	1,143 (95.2)	803 (95.5)	340 (94.7)	0.668
	Yes	57 (4.8)	38 (4.5)	19 (5.3)	
Preoperative arrhythmia (*n*%)	No	1,044 (87.0)	730 (86.8)	314 (87.5)	0.826
	Yes	156 (13.0)	111 (13.2)	45 (12.5)	
Chronic Obstructive Pulmonary Disease (COPD) (*n*%)	No	1,080 (90.0)	751 (89.3)	329 (91.6)	0.256
	Yes	120 (10.0)	90 (10.7)	30 (8.4)	
Preoperative cerebrovascular accident (*n*%)	No	1,049 (87.4)	741 (88.1)	308 (85.8)	0.311
	Yes	151 (12.6)	100 (11.9)	51 (14.2)	
Carotid artery stenosis (*n*%)	No	1,080 (90.0)	751 (89.3)	329 (91.6)	0.256
	Yes	120 (10.0)	90 (10.7)	30 (8.4)	
Renal artery stenosis (*n*%)	No	1,156 (96.3)	817 (97.1)	339 (94.4)	0.054
	Yes	44 (3.7)	24 (2.9)	20 (5.6)	
Peripheral vascular disease (*n*%)	No	869 (72.4)	603 (71.7)	266 (74.1)	0.436
	Yes	331 (27.6)	238 (28.3)	93 (25.9)	
Ventricular aneurysm (*n*%)	No	1,175 (97.9)	821 (97.6)	354 (98.6)	0.382
	Yes	25 (2.1)	20 (2.4)	5 (1.4)	
Preoperative Ejection Fraction (EF) [mean (SD)]		59.70 (8.67)	59.31 (8.97)	60.60 (7.86)	0.068
Preoperative left ventricular size [mean (SD)]		4.83 (0.55)	4.85 (0.56)	4.79 (0.52)	0.098
Preoperative Mechanical ventilation time [mean (SD)]		42.68 (66.50)	43.19 (70.07)	41.49 (57.35)	0.685
Intra-Aortic Balloon Pump (IABP) assistance before operation (*n*%)	No	1,133 (94.4)	789 (93.8)	344 (95.8)	0.212
	Yes	67 (5.6)	52 (6.2)	15 (4.2)	
Extracorporeal Membrane Oxygenation (ECMO) assistance before operation (*n*%)	No	1,198 (99.8)	841 (100.0)	357 (99.4)	0.163
	Yes	2 (0.2)	0 (0.0)	2 (0.6)	
Stroke (*n*%)	No	1,167 (97.3)	818 (98.1)	349 (99.2)	0.476
	Yes	33 (2.7)	23 (1.9)	10 (0.8)	

### Data preprocessing

The R package missForest version 1.4 was used to separately impute missing data points in the potential predictor variables for both the training and testing sample. Meanwhile, variables that had zero or nearly zero variance were also eliminated. To identify predictors with zero or near-zero variance, a general guideline is to consider the ratio of unique values to the sample size. In our study, this ratio was set to 10%.

### The collection of variables

A total of 33 preoperative clinical features were collected for the follow-up study, which were considered to be closely related to the prognosis of cardiovascular patients. These features included gender, age, Percutaneous Cardiology Intervention (PCI) history, smoking history, numbers of stenosed coronary vessels, New York Heart Association (NYHA) cardiac function classification, emergency or reoperation status, cardiac arrest occurrence, number of bridges, numbers of arterial and venous anastomoses, Cardiopulmonary bypass (CPB) and aortic cross-clamp times, cardiotonic drug usage, hypertension, diabetes mellitus and hyperlipidemia histories, preoperative creatinine value, hemodialysis, renal insufficiency, arrhythmia and Chronic Obstructive Pulmonary Disease (COPD) histories, preoperative cerebrovascular accident and carotid artery stenosis histories, renal artery stenosis and peripheral vascular disease histories, and ventricular aneurysm occurrence. Additionally, preoperative Ejection Fraction (EF), left ventricular size, mechanical ventilation time and Intra-Aortic Balloon Pump (IABP) or Extracorporeal Membrane Oxygenation (ECMO) assistance were investigated. Finally, postoperative complications, such as recurrent myocardial infarction, cerebrovascular accident, respiratory complications and renal dysfunction were documented. Patients in NYHA class III and IV experience marked limitations in physical activity or symptoms of heart failure even at rest. In our study, we recorded patients in NYHA class III and IV as “1” in Table 1. Medical records were reviewed to obtain all clinical data.

### Feature description

In our research, we used the Least Absolute Selection and Shrinkage Operator (LASSO) method to pre-select the features of candidates, followed by Partial Least Squares-Based Discriminant Analysis (PLS-DA) classification to select the best features. After being trained on training sets, the model's performance was assessed using test sets. The optimal hyperparameter of LASSO was selected using five-fold cross validation (*λ*). Furthermore, the risk factor ratio of each feature was calculated by indexing the Linear Regression (LR) coefficients, with a significance level of *P* < 0.05.

### Machine learning models

Seven machine learning models — XGBoost, logistic regression (LR), random forest (RF), Decision Tree (DT), Support Vector Classification (SVC), Gradient boosting (GB) and K-Nearest-Neighbors (KNN) — were used to develop the predictive models, which has been explained in our previous study (11). *Python* (3.8.5) was used to implement all machine learning models using scikit-learn (1.0.2) packages.

### Evaluation measure

In order to assess the effectiveness of various machine learning techniques, the *F1*-Score was employed as a means of gauging the precision and recall balance in binary classification:

$$F1=2*\frac{\mathrm{Precision}*\;\;\mathrm{Recall}}{\mathrm{Precision}+\mathrm{Recall}}$$

Additionally, additional standard measures of performance were calculated, such as Accuracy, Precision, Recall, and AUC.

### Machine learning explainable tool

SHAP, also known as SHapley Additive exPlanation, is a comprehensive method used to interpret machine learning models by offering explanations for individual predictions. By utilizing SHAP Values, scientists have the ability to measure the influence of each characteristic on the result and ascertain whether it had a beneficial or detrimental effect on the forecast. This provides an effective way to uncover the underlying mechanisms behind the model and analyze its performance in further detail. The interpretation of model predictions was done by computing SHAP values using the shap Python package.

### Statistical analyses

This study used a machine learning approach to analyze data and predict outcomes for various problems. The performance of the machine learning methods was evaluated using the *R* (4.2.2) and *Python* (Version 3.8.5) software packages, and statistical analysis was conducted. The AUC, which stands for the area below the ROC (relative operating characteristic) curve, was utilized for evaluation.

In this study, we employed independent-samples *T*-test, Mann–Whitney *U*-test, and Chi-Square test to statistically examine the preoperative and intraoperative conditions of patients, taking into account the various variable types and their collective distribution. Statistical significance was attributed to *P* values below 0.05.The data were expressed as mean ± s.e.m.

## Results

### Patient characteristics

**Figure 1:**
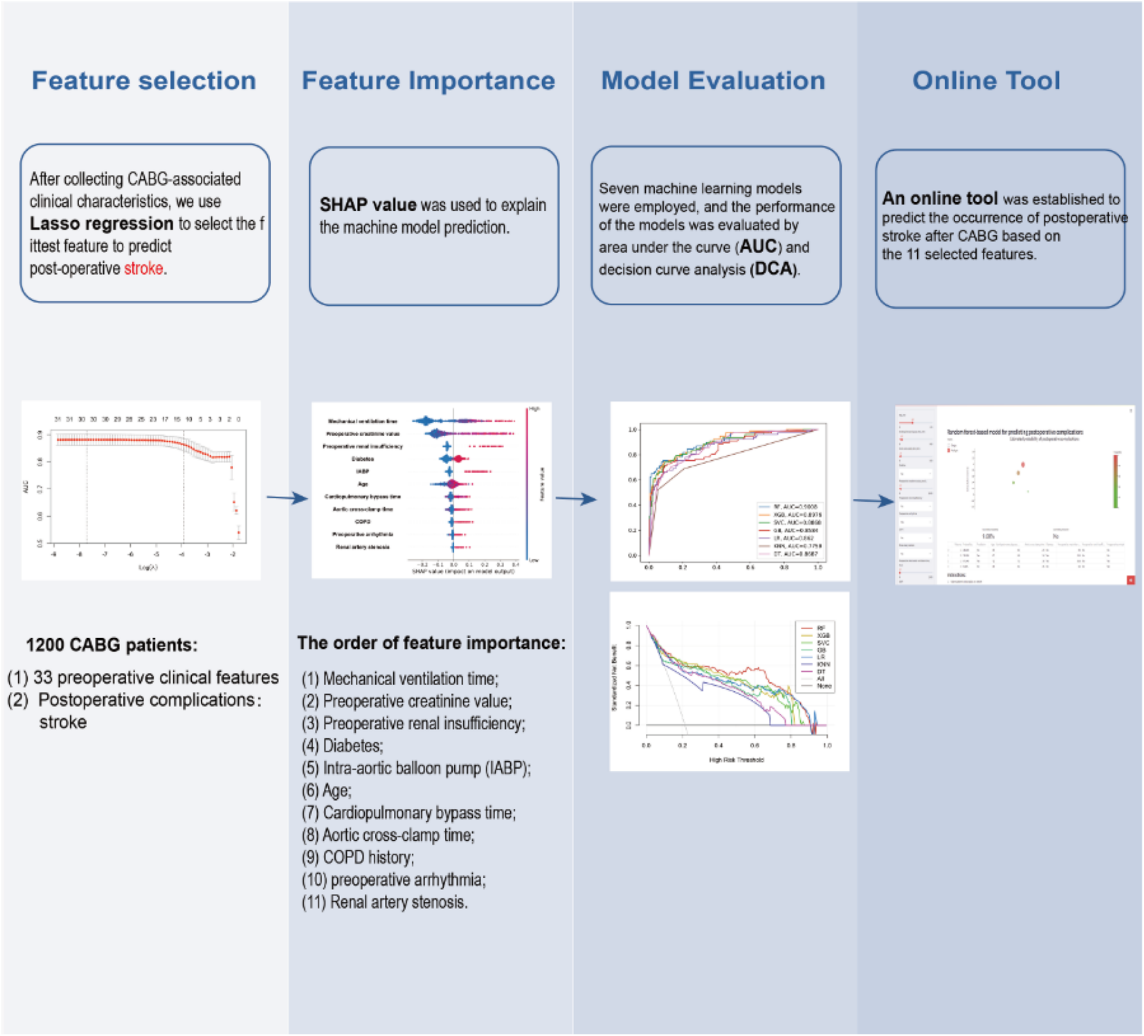
Flow chart for the study.

The study flow diagram was displayed in Figure 1. A study involving 1,200 patients was conducted to evaluate the impact of 33 clinical characteristics on postoperative complications. Of these, 70% were randomly assigned to a training set (841 instances), while the remaining 30% constituted the test set (359 instances). Table 1 displays the attributes of the patients. The average age of the participants in the study was 60.26 years. 910 (75.8%) of the patients were men, and 153 (12.8%) patients were in NYHA class III and IV. 12.8% of patients had underwent percutaneous coronary intervention (PCI). Among the cohort, 35 (2.9%) of patients died in hospital, 16 (1.3%) had emergency operation for acute chest pain, 10 (0.8%) had undergone chest re-exploration, and 2 (0.2%) had undergone extracorporeal membrane oxygenation (ECMO).

### Feature selection

**Figure 2:**
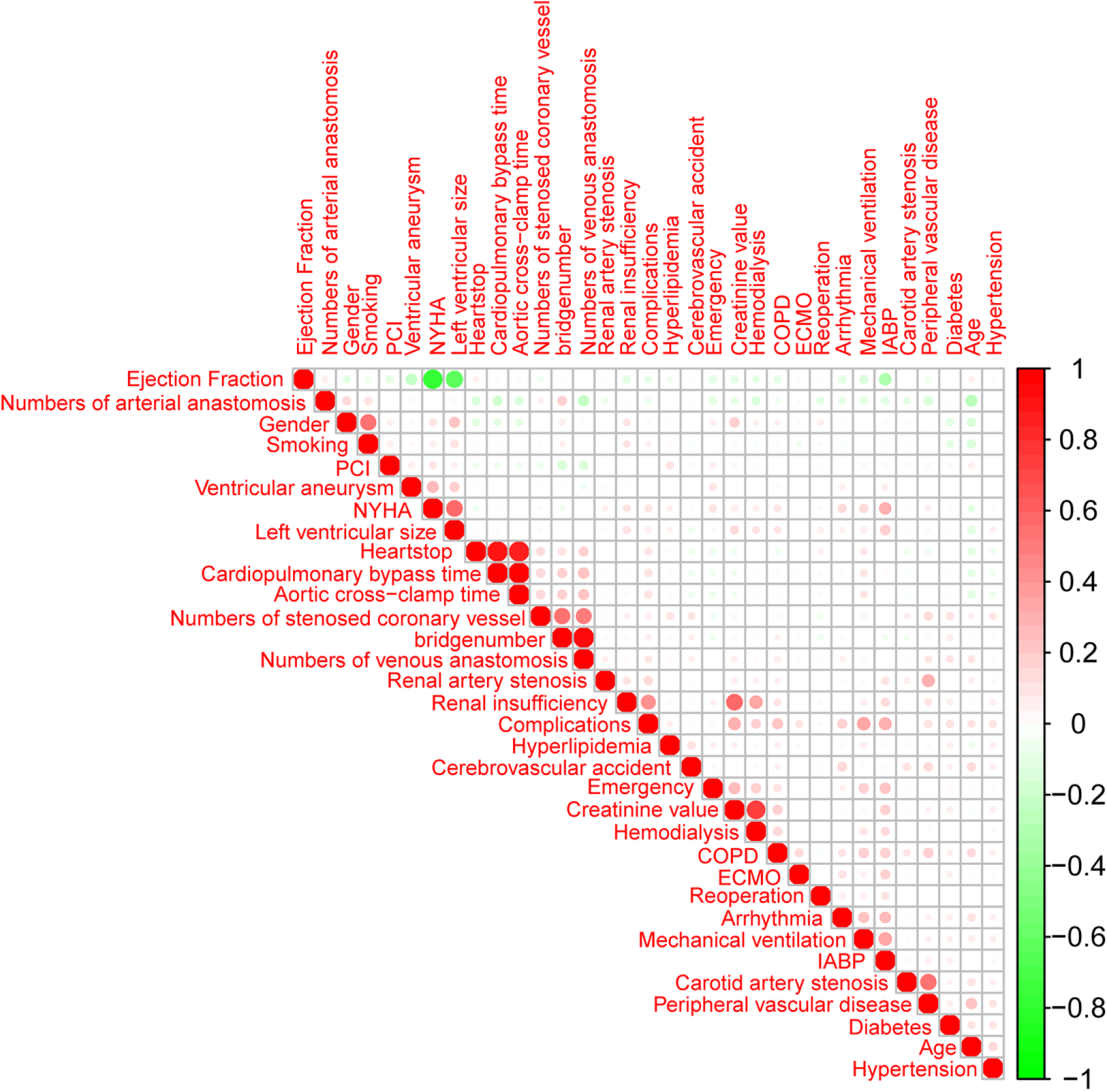
An analysis of correlations between variables. Red is a coefficient of 1, white is a coefficient of 0, and green is a coefficient of −1.

**Figure 3:**
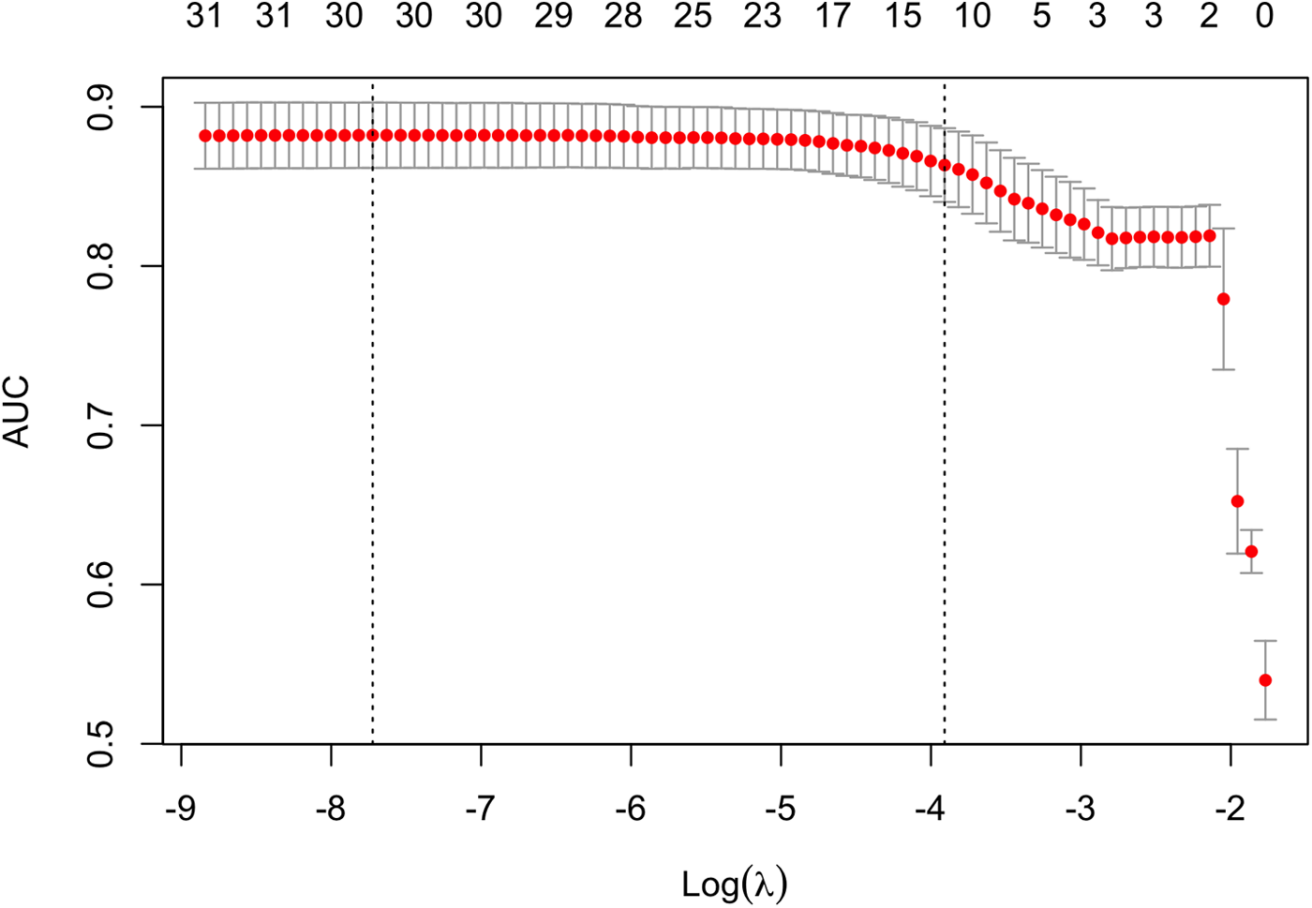
The Least Absolute Shrinkage and Selection Operator (LASSO) algorithm for feature selection. In the LASSO model, 5-fold cross-validation through minimum criteria was used based on the binomial deviance metrics (the *y*-axis).

**Figure 4:**
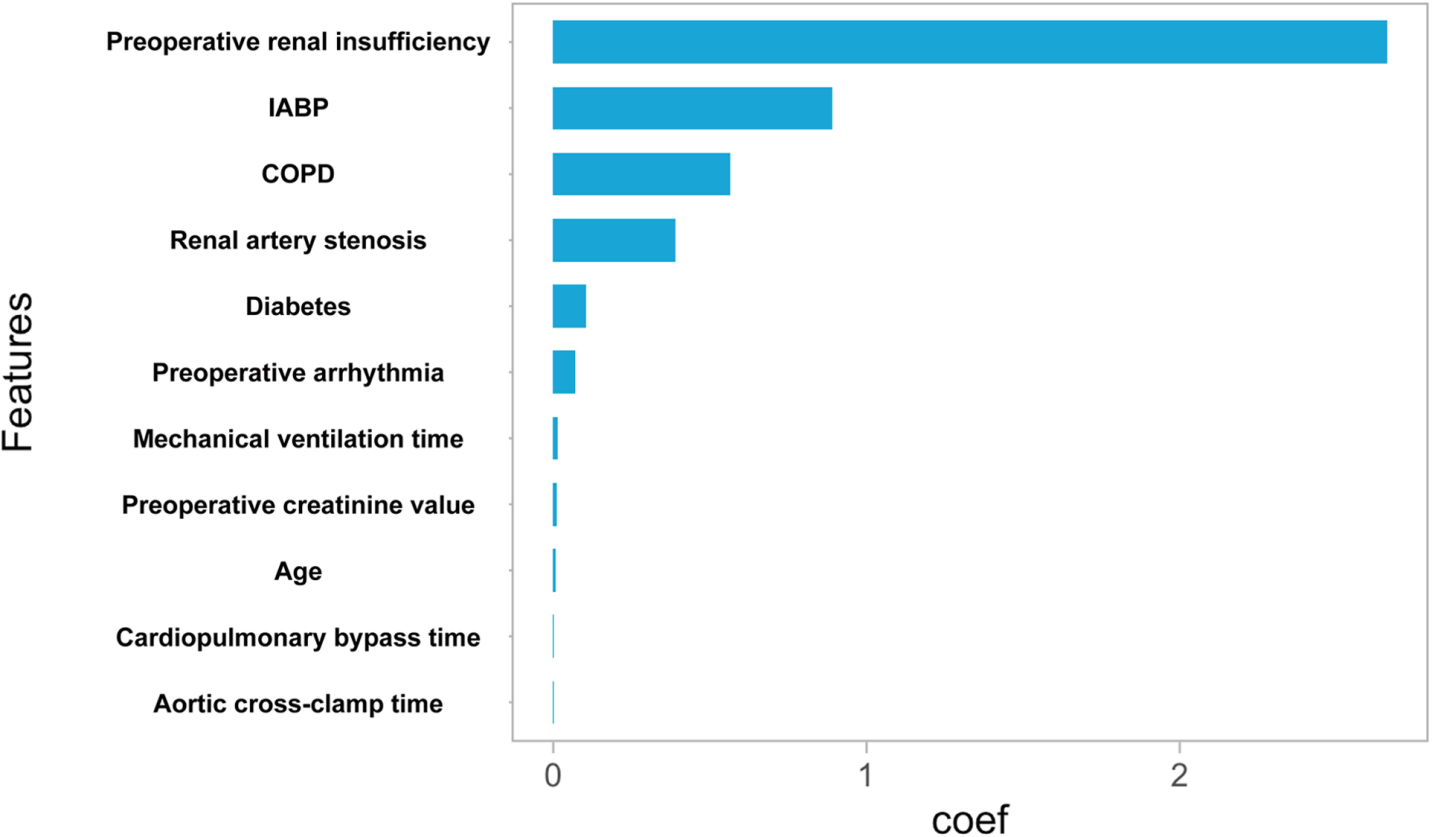
Feature importance using lasso feature selection technique. Rank of importance of selected features according to Lasso algorithm.

**Figure 5:**
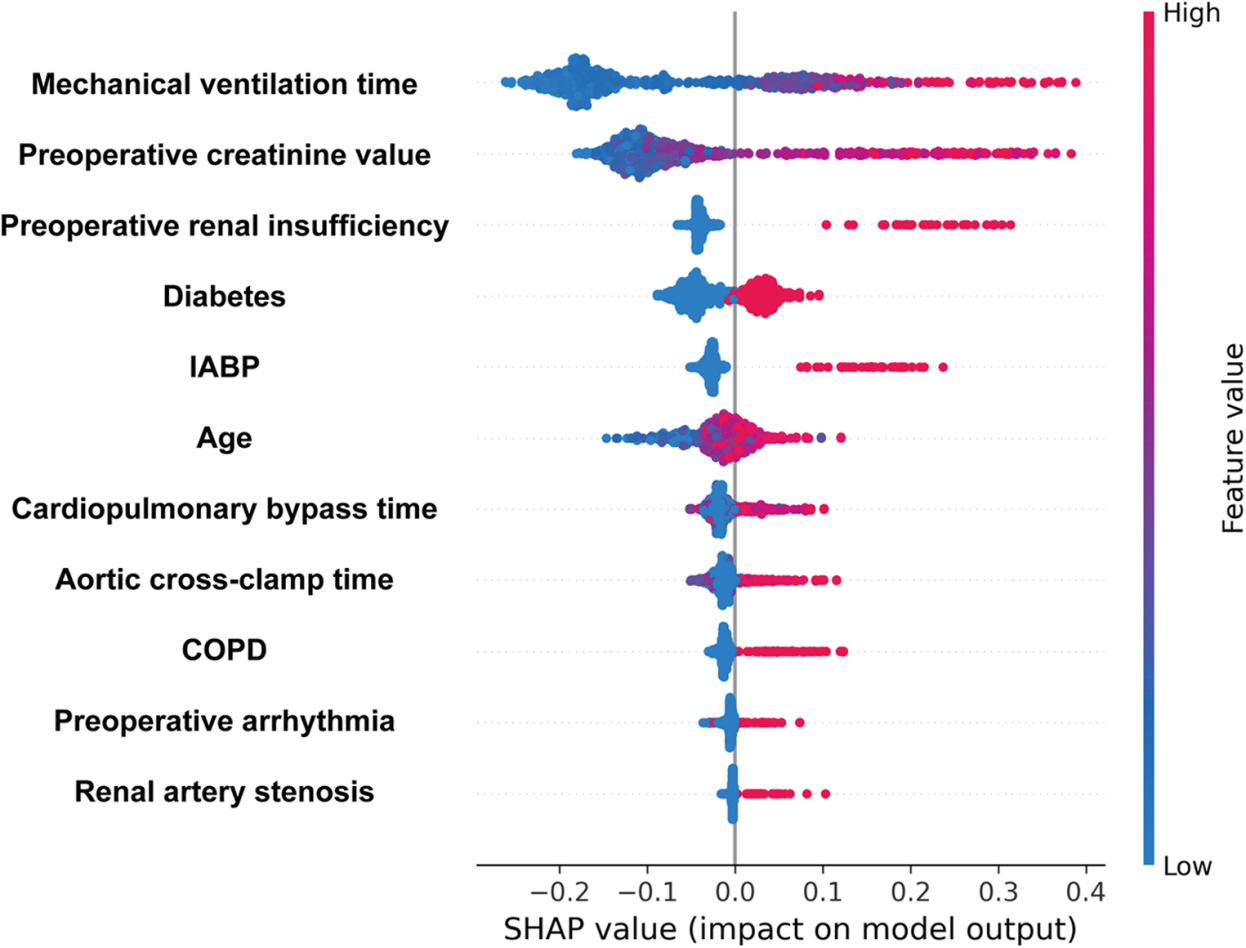
Variable importance measured by Shapley Additive Explanation (SHAP) values. A point's color represents its feature value, and its position on the *X*-axis represents its SHAP value. Features are ranked according to their SHAP value magnitudes.

The training set and test set contain 33 variables with a *P* value greater than 0.05, suggesting no significant distinction between the two groups. The detailed flow chart of this study is illustrated in Figure 1. Initially, we conducted a correlation analysis on 33 chosen clinical variables, and the comprehensive outcomes are presented in Figure 2. Variables with high correlation were removed to reduce multicollinearity, ensuring that each selected feature contributed uniquely to the predictive model without redundant information. We removed 2 variables (Heart arrest and Left ventricular size) based on high correlation with other items, 31 variables remained. Features like mechanical ventilation time and preoperative creatinine value are directly linked to increased stroke risk due to their roles in cerebral perfusion and metabolic imbalance. Next, we employed the LASSO algorithm to identify the minimum threshold (*λ*) and subsequently chose 11 significant attributes for additional investigation (Figure 3). Meantime, the 11 features we selected related to postoperative stroke are as follows: preoperative renal insufficiency, intra-aortic balloon pump (IABP), chronic obstructive pulmonary disease (COPD), renal artery stenosis, diabetes, preoperative arrhythmia, mechanical ventilation time, preoperative creatinine value, age, cardiopulmonary bypass time, aortic cross-clamp time (Figure 4). SHAP is a method to solve the interpretability of models. All samples are ranked based on their SHAP values. In order of importance, these variables are: (1) mechanical ventilation time; (2) preoperative creatinine value; (3) preoperative renal insufficiency; (4) diabetes; (5) the use of an intra-aortic balloon pump (IABP); (6) age; (7) Cardiopulmonary bypass time; (8) Aortic cross-clamp time; (9) COPD history; (10) preoperative arrhythmia; and (11) Renal artery stenosis (Figure 5).

### Performance of prediction models for postoperative complications in patients with coronary artery bypass grafting

**Figure 6:**
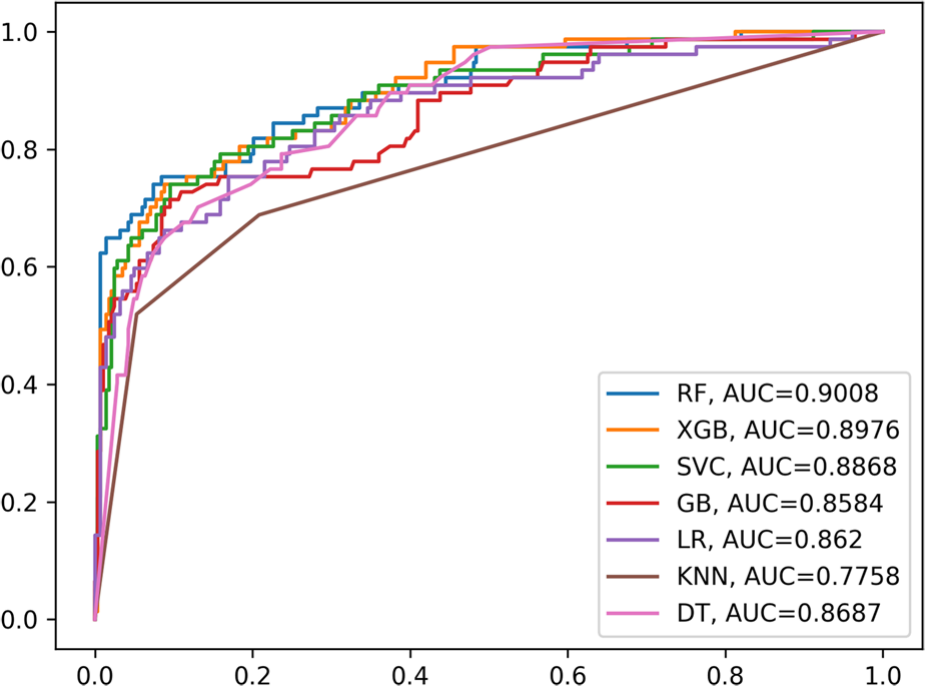
The ROC curves of six machine learning models and the LR model.

The results of the receiver operating characteristics (ROC) evaluation of six machine learning models and logistic regression, each trained on eleven identified variables, are shown in Figure 6. The RF model demonstrated the highest AUC score of 0.9008, with the XGB model following closely at 0.8976, and the SVC model achieving a score of 0.8868.The GB model, LR model, KNN model, and DT model exhibited AUC scores of 0.8584, 0.8620, 0.7758, and 0.8687, respectively. The results are summarized in Table 2.

**Table 2:** Performance of machine learning models.

Models	Accuracy	Precision	Recall	F1	AUC
RF	0.900821	0.690476	0.753247	0.720497	0.900831
XGboost	0.897632	0.705128	0.714286	0.709677	0.897572
SVC	0.886886	0.71831	0.662338	0.689189	0.886788
GB	0.858421	0.67521	0.701299	0.687898	0.858405
LR	0.862054	0.666667	0.649351	0.657895	0.862007
KNN	0.775834	0.727273	0.519481	0.606061	0.775825
DT	0.868797	0.593407	0.701299	0.642857	0.868707

Accuracy, Precision, Recall and F1 indicate the classification performance. The F1 score is the harmonic mean of precision and recall while the accuracy is the recall. (RF, random forest; SVC, support vector machine; GB, gradient boosting; LR, logistic regression; KNN, K-nearest-neighbors; DT, decision tree; AUC, area under the curve).

**Figure 7:**
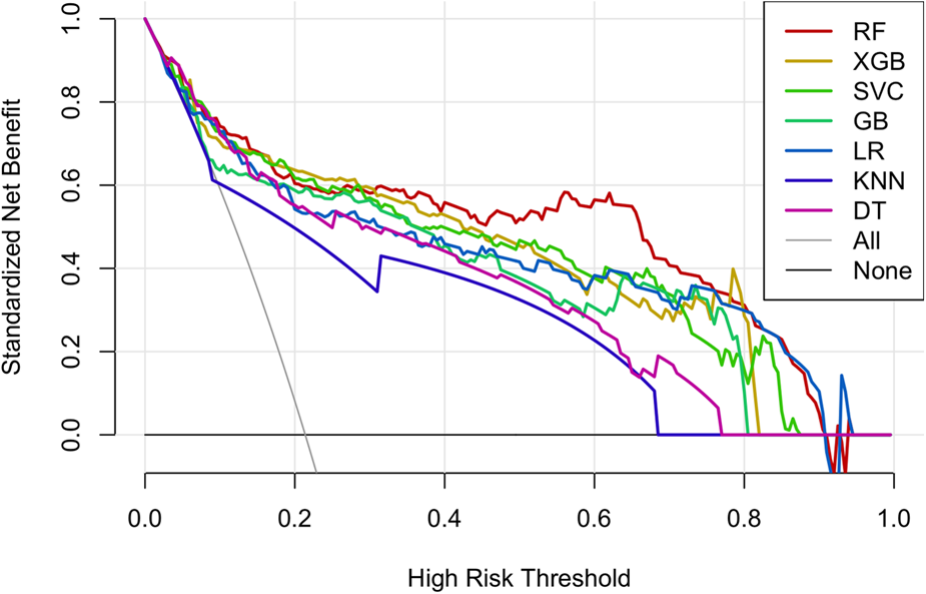
The DCA (decision curve analysis) curve of the six machine learning models and the LR model.

Decision Curve Analysis (DCA) was used to compare the reliability of the six machine learning models and logistic regression model in predicting postoperative complications after coronary artery bypass grafting. The results suggest that the machine learning models displayed well predictive ability, with the RF model performing best (Figure 7). The curves of each model were higher than the two extreme cases, showing that their net benefit was positively associated with increased threshold probability. Therefore, even though ROC curves are conventional methods for determining the ease of use of diagnostic methods, DCA could be a suitable alternative as it takes into account more factors.

**Figure 8:**
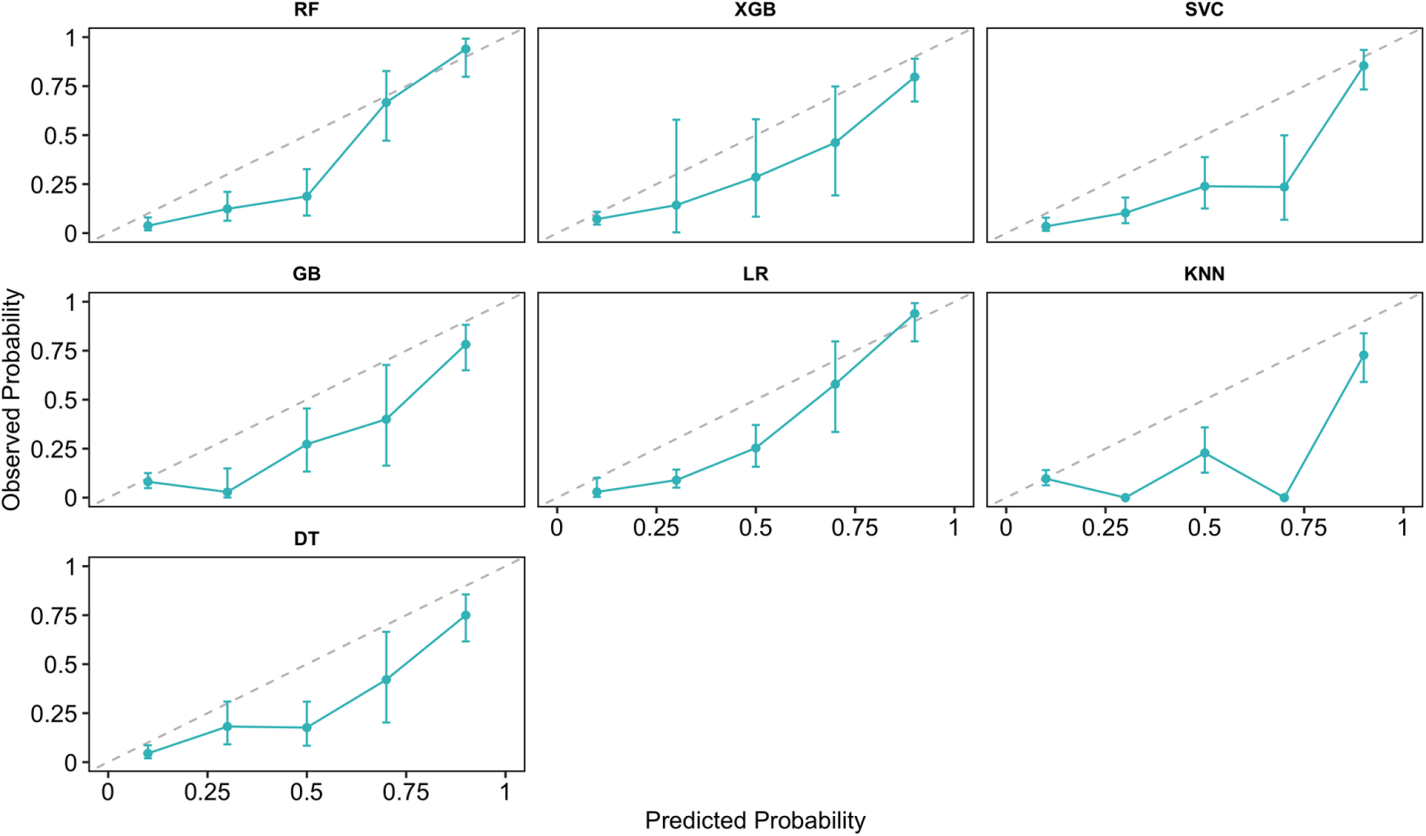
The calibration curves for the six machine learning models and the LR model.

The strength of the model outcomes was additionally backed by the responsiveness of every machine model. The predicted probability of the six machine learning models and logistic regression model is depicted in Figure 8.

### Online prediction tool

**Figure 9:**
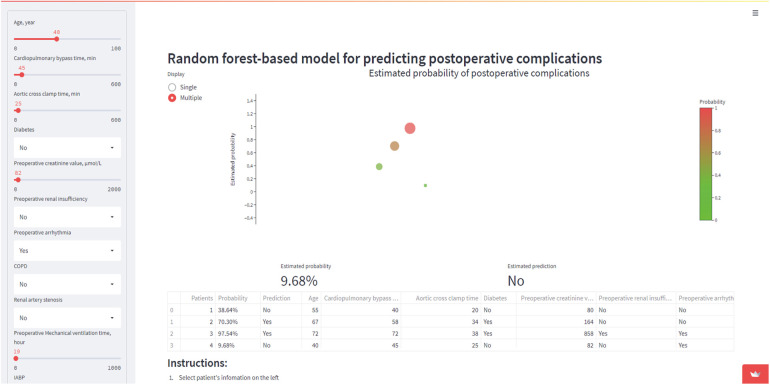
Four fictitious case scenarios were generated by the online prediction tool. Use 11 key variables to predict postoperative stroke.

We built an online interpretable RF machine learning model to predict the occurrence of postoperative stroke after coronary artery bypass grafting based on eleven selected features (https://whuh-ml-complications-app-jog3zl.streamlitapp.com/). The RF model was built with the whole training dataset, taking 45 min to build the model and 50 ms for producing prediction results after inputting the selected variables by using our platform. A prediction tool was used to generate results for four scenarios in Figure 9.

## Discussion

Coronary artery disease (CAD) is caused by atherosclerosis and other risk factors involved in coronary heart disease (12). Surgery is considered to be the most effective modality in the treatment of CHD. And The most common form of cardiac surgery is CABG, which is widely used to treat coronary heart disease (13). Although previous studies have excellent results upon employing conventional CABG, postoperative stroke also occurs in about 1.3% of CABG patients (14). In addition, in current study, patients with on-pump CABG and off-pump CABG had similar incidence of stroke during follow-up (15).

Meanwhile, numerous research has demonstrated that the presence of postoperative stroke has a significant adverse effect on patients' short- and long-term prognoses, the duration of hospital stay, lifespan, and quality of life post-discharge are all considered (16). After cardiac surgery, the risk of postoperative stroke remains relatively high. It is urgent to improve the existing treatment strategy.

Approximately half of all CABG patients have aortic atherosclerosis, therefore CABG surgery carries a high risk of stroke (14). Otherwise, compared with the general US population, CHD mortality rates were lower, but stroke mortality rates were higher (17). Therefore, it is imperative to identify high-risk stroke groups early and implement prevention, to make patients benefit from brain protection, we can take a more active approach.

The aim of this research is to identify the factors that contribute to postoperative stroke by examining the preoperative and intraoperative variables, and to develop a mathematical framework for postoperative stroke. In our study, Mechanical ventilation time, preoperative creatinine value, preoperative renal insufficiency, diabetes, the use of an intra-aortic balloon pump (IABP), age, Cardiopulmonary bypass time, Aortic cross-clamp time, Chronic Obstructive Pulmonary Disease (COPD) history, preoperative arrhythmia and Renal artery stenosis were the key independent predictors.

Previous studies have established cardiac surgery patients who had a longer duration of mechanical ventilation will extend the length of hospital stay and the mortality (18). And longer mechanical ventilation time could exacerbate brain injury. Otherwise, a previous diabetic history was found to increase the risk of stroke postoperatively. Diabetes mellitus was the second most dominant factor to be associated with incidence of stroke (19). As for cardiopulmonary bypass time and aortic cross-clamp time, previous studies suggested that shorter or avoiding cardiopulmonary bypass in patients will reduced the incidence of postoperative stroke (20). Meantime, incident stroke in Chronic obstructive pulmonary disease (COPD) also can accelerate and further increase mortality risk (21). Also, Arrhythmias such as atrial fibrillation can cause cardiac function decline and increase the risk of cerebral stroke in patients (22). Of course, age is also a major risk factor for stoke (23). In general, the rate of incidence is higher among elderly patients with poor general function compared to the general population. These conclusions all are consistent with our results.

To mitigate the occurrence of postoperative strokes, our objective is to implement enhanced brain-protective strategies, encompassing intraoperative cerebral oximetry monitoring, elevated cerebral perfusion pressures, preoperative arrhythmia detection and management, long-term preservation of renal function, and minimizing the duration of cardiopulmonary bypass.

Our study also has a few limitations. First, the data used in this study were collected from a single center with no external validation, and this may introduce a possible regional bias. Second, our preoperative risk factors and postoperative complications were not fully accounted for. Although we have tried our best to collect all the available information, some data could still be missing. In addition, machine learning approaches are known to greatly benefit from large sample size. However, in our study, 1,200 patients with 33 clinical characteristics were indeed smaller, which may potentially lead to overfitting. Therefore, we used cross-validation and built simpler model to avoid overfitting. Also, we perform model selection on a per-variable basis, avoiding the possibility of overfitting that the more flexible model presents. Further more detailed and advanced method research is needed to address these limitations and better understand the findings of this study.

As a result, several preoperative factors were identified, including renal insufficiency, arrhythmia, diabetes, and COPD, as well as an intraoperative factor, CPB time, Mechanical ventilation time, and aortic cross-clamp time were strong predictors of postoperative stroke in CABG patients. To predict these factors, we construct a Logistic regression model and six machine learning models. Random forest model present best and construct an online predict website based on this model, and this AI website can be used as a clinical decision support systems, to help the clinicians in decision making.

## Data Availability

The original contributions presented in the study are included in the article/Supplementary Material, further inquiries can be directed to the corresponding author.

## References

[1] Gilboa SM Devine OJ Kucik JE Oster ME Riehle-Colarusso T Nembhard WN Congenital heart defects in the United States: estimating the magnitude of the affected population in 2010. Circulation. (2016) 134:101–9. 10.1161/CIRCULATIONAHA.115.019307 27382105 PMC4942347

[2] Laakso M. Heart in diabetes: a microvascular disease. Diabetes Care. (2011) 34(2):S145–9. 10.2337/dc11-s209 21525446 PMC3632152

[3] Toribio M Fitch KV Stone L Zanni MV Lo J de Filippi C Assessing statin effects on cardiovascular pathways in HIV using a novel proteomics approach: analysis of data from INTREPID, a randomized controlled trial. Ebiomedicine. (2018) 35:58–66. 10.1016/j.ebiom.2018.08.039 30174281 PMC6156703

[4] Benstoem C Stoppe C Liakopoulos OJ Ney J Hasenclever D Meybohm P Remote ischaemic preconditioning for coronary artery bypass grafting (with or without valve surgery). Cochrane Database Syst Rev. (2017) 5:D11719. 10.1002/14651858.CD011719.pub3 PMC6481544 28475274

[5] Zembala M Michler RE Rynkiewicz A Huynh T She L Lubiszewska B Clinical characteristics of patients undergoing surgical ventricular reconstruction by choice and by randomization. J Am Coll Cardiol. (2010) 56:499–507. 10.1016/j.jacc.2010.03.054 20670761 PMC2936491

[6] Al-Ruzzeh S Ambler G Asimakopoulos G Omar RZ Hasan R Fabri B Off-pump coronary artery bypass (OPCAB) surgery reduces risk-stratified morbidity and mortality: a United Kingdom multi-center comparative analysis of early clinical outcome. Circulation. (2003) 108(1):I1–8. 10.1161/01.cir.0000087440.59920.a1 12970199

[7] Eagle KA Guyton RA Davidoff R Ewy GA Fonger J Gardner TJ ACC/AHA guidelines for coronary artery bypass graft surgery: executive summary and recommendations: a report of the American college of cardiology/American heart association task force on practice guidelines (committee to revise the 1991 guidelines for coronary artery bypass graft surgery). Circulation. (1999) 100:1464–80. 10.1161/01.CIR.100.13.1464 10500052

[8] Goh KH Wang L Yeow A Poh H Li K Yeow JJL Artificial intelligence in sepsis early prediction and diagnosis using unstructured data in healthcare. Nat Commun. (2021) 12:711. 10.1038/s41467-021-20910-4 33514699 PMC7846756

[9] Agha R Abdall-Razak A Crossley E Dowlut N Iosifidis C Mathew G STROCSS 2019 guideline: strengthening the reporting of cohort studies in surgery. Int J Surg. (2019) 72:156–65. 10.1016/j.ijsu.2019.11.002 31704426

[10] Mathew G Agha R Albrecht J Goel P Mukherjee I Pai P STROCSS 2021: strengthening the reporting of cohort, cross-sectional and case-control studies in surgery. Int J Surg. (2021) 96:106165. 10.1016/j.ijsu.2021.106165 34774726

[11] Wang K Yan LZ Li WZ Jiang C Wang NN Zheng Q Comparison of four machine learning techniques for prediction of intensive care unit length of stay in heart transplantation patients. Front Cardiovasc Med. (2022) 9:863642. 10.3389/fcvm.2022.863642 35800164 PMC9253610

[12] Zhou J Shao G Chen X Yang X Huang X Peng P miRNA 206 and miRNA 574-5p are highly expression in coronary artery disease. Biosci Rep. (2015) 36:e295. 10.1042/BSR20150206 PMC4748332 26685009

[13] Pan XX Ruan CC Liu XY Kong LR Ma Y Wu QH Perivascular adipose tissue-derived stromal cells contribute to vascular remodeling during aging. Aging Cell. (2019) 18:e12969. 10.1111/acel.12969 31087498 PMC6612678

[14] Ozmen R Bozguney M Tekin AI Eroglu T Tuncay A. Impact of single versus double clamp technique on blood lactate levels and postoperative complications in coronary artery bypass Surgery. Braz J Cardiovasc Surg. (2022) 37:55–64. 10.21470/1678-9741-2020-0025 33656827 PMC8973127

[15] Wang S Ran Y Cheng S Lyu Y Liu J. Determinants and clinical outcomes of stroke following revascularization among patients with reduced ejection fraction. Brain Behav. (2023) 13:e2927. 10.1002/brb3.2927 36860139 PMC10097158

[16] Raman N Al-Robaidi K Jadhav A Thirumala PD. Perioperative stroke and readmissions rates in noncardiac non-neurologic surgery. J Stroke Cerebrovasc Dis. (2020) 29:104792. 10.1016/j.jstrokecerebrovasdis.2020.104792 32280000

[17] Howard BV Metzger JS Koller KR Jolly SE Asay ED Wang H All-cause, cardiovascular, and cancer mortality in western Alaska native people: western Alaska tribal collaborative for health (WATCH). Am J Public Health. (2014) 104:1334–40. 10.2105/AJPH.2013.301614 24754623 PMC4056205

[18] Cao CC Chen DW Li J Ma MQ Chen YB Cao YZ Community-acquired versus hospital-acquired acute kidney injury in patients with acute exacerbation of COPD requiring hospitalization in China. Int J Chron Obstruct Pulmon Dis. (2018) 13:2183–90. 10.2147/COPD.S164648 30140150 PMC6054768

[19] Xu X Mishra GD Dobson AJ Jones M. Progression of diabetes, heart disease, and stroke multimorbidity in middle-aged women: a 20-year cohort study. PLoS Med. (2018) 15:e1002516. 10.1371/journal.pmed.1002516 29534066 PMC5849280

[20] Jia H Huang B Kang L Lai H Li J Wang C Preoperative and intraoperative risk factors of postoperative stroke in total aortic arch replacement and stent elephant trunk implantation. Eclinicalmedicine. (2022) 47:101416. 10.1016/j.eclinm.2022.101416 35518120 PMC9062417

[21] Kim YR Hwang IC Lee YJ Ham EB Park DK Kim S. Stroke risk among patients with chronic obstructive pulmonary disease: a systematic review and meta-analysis. Clinics. (2018) 73:e177. 10.6061/clinics/2018/e177 29723340 PMC5910631

[22] Cai W Hu J Wang H Chen S Zhu G Chen X. Valve surgery in combination with cryoablation in the treatment of atrial fibrillation. Pak J Med Sci. (2018) 34:1402–7. 10.12669/pjms.346.15537 30559793 PMC6290212

[23] Aissa I Elkoundi A Andalousi R Benakrout A Chlouchi A Moutaoukil M Unusual localization of bleeding under acenocoumarol: spinal subdural hematoma. Int J Surg Case Rep. (2019) 59:15–8. 10.1016/j.ijscr.2019.04.053 31100481 PMC6522769

